# Polo-like kinase 1 promotes pulmonary hypertension

**DOI:** 10.1186/s12931-023-02498-z

**Published:** 2023-08-19

**Authors:** Rongrong Chen, Hongfei Wang, Cuiting Zheng, Xiyu Zhang, Li Li, Shengwei Wang, Hongyu Chen, Jing Duan, Xian Zhou, Haiyong Peng, Jing Guo, Anchen Zhang, Feifei Li, Wang Wang, Yu Zhang, Jun Wang, Chen Wang, Yan Meng, Xinling Du, Hongbing Zhang

**Affiliations:** 1 State Key Laboratory of Common Mechanism Research for Major Diseases, Haihe Laboratory of Cell Ecosystem, Department of Physiology, Institute of Basic Medical Sciences and School of Basic Medicine, Peking Union Medical College and Chinese Academy of Medical Sciences, Beijing, China; 2 Department of Cardiac Surgery, Institute of Cardiovascular Disease, Union Hospital, Tongji Medical College, Huazhong University of Science and Technology, Wuhan, China; 3 Department of Pathology, Beijing Lab for Cardiovascular Precision Medicine, Key Laboratory of Medical Engineering for Cardiovascular Disease, Capital Medical University, Beijing, China; 4 Department of Physiology, Capital Medical University, Beijing, China; 5 State Key Laboratory of Molecular Oncology, National Cancer Center/National Clinical Research Center for Cancer/Cancer Hospital, Chinese Academy of Medical Sciences and Peking Union Medical College, Beijing, China

**Keywords:** Pulmonary hypertension, Hypoxia, Polo-like kinase 1, HIF1α, RELA/p65

## Abstract

**Background:**

Pulmonary hypertension (PH) is a lethal vascular disease with limited therapeutic options. The mechanistic connections between alveolar hypoxia and PH are not well understood. The aim of this study was to investigate the role of mitotic regulator Polo-like kinase 1 (PLK1) in PH development.

**Methods:**

Mouse lungs along with human pulmonary arterial smooth muscle cells and endothelial cells were used to investigate the effects of hypoxia on PLK1. Hypoxia- or Sugen5416/hypoxia was applied to induce PH in mice. *Plk1* heterozygous knockout mice and PLK1 inhibitors (BI 2536 and BI 6727)-treated mice were checked for the significance of PLK1 in the development of PH.

**Results:**

Hypoxia stimulated PLK1 expression through induction of HIF1α and RELA. Mice with heterozygous deletion of *Plk1* were partially resistant to hypoxia-induced PH. PLK1 inhibitors ameliorated PH in mice.

**Conclusions:**

Augmented PLK1 is essential for the development of PH and is a druggable target for PH.

## Introduction

Pulmonary hypertension (PH) is a severe disorder of pulmonary vasculature. Excessive proliferation of vascular cells, increased extracellular matrix deposition, and accumulation of inflammatory cells collectively cause high pulmonary pressure and eventually leads to right heart failure even death [1, 2]. Hypoxia is a well-documented risk factor for PH [1]. For these PH patients associated with hypoxic lung disorders, oxygen therapy is the regimen that might reduce mortality. However, the survival advantage is not obvious unless patients receive more than 500 days of oxygen therapy (15 h/day). Although prostanoids and endothelin receptor antagonists are effective in the treatment of certain types of PH, those therapeutics also exacerbate ventilation-perfusion mismatch and increase hypoxemia in these patients [3–5]. Novel therapeutic strategies are thus warranted for a subset of PH patients.

Hypoxia-inducible factors (HIFs) are key transcription factors to mediate adaptive responses to hypoxia. HIFs are made up of an O_2_-sensitive α-subunit (mainly HIF1α and HIF2α) and a constitutively expressed β-subunit [6]. HIF1α is elevated because of its reduced ubiquitylation and decreased proteasome-mediated degradation under hypoxic conditions [7]. It translocates to the nucleus and dimerizes with the β subunit to stimulate plethora of gene expressions. In response to chronic hypoxia, induced HIF1α contributes to the vascular remodeling and promotes PH [6]. Development of novel therapeutic strategies may rely on dissection of the events between hypoxia induction of HIF1α and PH.

Hypoxia activates nuclear factor-kappa B (NF-κB) [8] which is a critical transcription factor involved in the activation of pro-inflammatory response, cell proliferation and tumorigenesis [9]. NF-κB has also been implicated in the progression of PH. Pulmonary arterial lesions of PH patients show increased NF-κB activation [10]. Hypoxia induces RELA/p65, a subunit member of NF-κB, nuclear translocation and accumulation [11]. In the nucleus, RELA/p65 interacts with HIF-1α to boost gene transcription [12]. HIF1α also promotes RELA transcription under hypoxia [13]. On the other hand, NF-κB is involved in transactivation of HIF1α transcription. Therefore, there are cross-talks between HIF1α and NF-κB [14].

As a major mitotic kinase, Polo-like kinase 1 (PLK1) promotes cell proliferation largely through stimulation of centrosome maturation, mitotic entry, spindle assembly, and cytokinesis as well as maintenance of genomic stability [15–19]. PLK1 has been shown to regulate smooth muscle contraction, centrosome maturation and apoptosis [20–23]. BI 2536 and BI 6727 (volasertib) are the lead agents of Plk1 inhibitors. The mitotic arrest caused by BI 2536 or BI 6727 is followed by a surge in apoptosis [24, 25]. BI 2536 and BI 6727 have been tested in clinical trials of multiple tumor types including lung cancers [24, 26, 27]. Moreover, Food and Drug Administration (FDA) has awarded BI 6727 the Breakthrough Therapy status after significant benefit was observed in acute myeloid leukemia patients [25, 28]. Since PH is featured with excessive cell proliferation and impaired cell death [2, 29–32], PLK1 is a potential target for intervention of this obstructive vasculopathy.

In this study, we first noticed hypoxic induction of PLK1. We then explored the potential involvement of HIF1α and NF-κB in induction of PLK1 expression. In addition, we investigated the putative role of PLK1 in the development of hypoxia-mediated and combined Sugen5416/hypoxia-induced (SuHx) mouse PH. Lastly, we tested the efficacy of targeting PLK1 for the treatment of PH.

## Methods

### Reagents and materials

Primary antibodies used for immunoblotting were as follows: mouse monoclonal PLK1 antibody (35-206, Millipore, Billerica, MA, USA); rabbit polyclonal α-SMA (55135-1-AP, Proteintech, Wuhan, China); mouse polyclonal GAPDH antibody (#2118, Cell Signaling Technology, Danvers, MA, USA); rabbit polyclonal HIF1α antibody (NB100-134, Novus Biologicals, Littleton, CO, USA); rabbit polyclonal HIF1α antibody (ab2185, Abcam, Cambridge, MA, USA); rabbit polyclonal alpha-smooth muscle-actin antibody (ab5694, Abcam); mouse monoclonal alpha-smooth muscle-actin antibody (A2547, Sigma-Aldrich, St Louis, MO, USA); mouse monoclonal ACTB/β-Actin antibody (sc-8432, Santa Cruz Biotechnology, Dallas, TX, USA); rabbit polyclonal RELA/p65 antibody (#3034, Cell Signaling Technology). Secondary antibodies used were as follows: FITC-conjugated goat anti-rabbit IgG (sc-2012, Santa Cruz Biotechnology), HRP-labeled goat anti-mouse IgG (sc-2005, Santa Cruz Biotechnology), HRP-labeled goat anti-rabbit IgG (sc-2004, Santa Cruz Biotechnology).

Lipofectamine 2000 (11668-019) was from Invitrogen (Carlsbad, CA, USA). DMEM (SH30022.01B) and FBS (SV30087.02) were from HyClone (Logan, UT, USA). Chemiluminescence (NC15079) was from Thermo Scientific (Waltham, MA, USA). MICROFIL (MV-122) was from Flow Tech Inc (Carver, MA, USA). For cell culture, PLK1 inhibitor BI 2536 (Axon 1129) was from Axon Medchem (Groningen, Netherlands). For animal experiments, BI 2536 was synthesized by WuXi AppTec (Shanghai, China). BI 6727 (S2235) was from Selleck (Houston, TX, USA). Ketamin (100 mg/mL) was from Fujian Gutian Pharmaceuticals (Gutian, China) and fentanyl (20 mg/mL) was from Yichang Ren Fu Pharmaceuticals (Yichang, China). Trizol reagent (15,596–018, Invitrogen), PrimeScript RT Reagent Kit (DRR037A, TaKaRa, Kyoto, Japan), TransStart Green qPCR SuperMix (AQ131-03, TransGen Biotech, Beijing, China) and SYBR PremixEx Taq (RR420A, TaKaRa) were used for Quantitative real-time PCR on an IQ5 (Bio-Rad Laboratories, Hercules, CA, USA).

PLK1 inhibitor BI 2536 was prepared as a 100 μM stock solution in sterile water and diluted in DMEM tissue culture medium at the indicated concentrations immediately before use. BI 6727 was prepared as a 200 μM stock solution in DMSO and diluted in DMEM tissue culture medium at the indicated concentrations immediately before use.

### Cell culture

Human pulmonary arterial smooth muscle cells (hPASMCs) and human pulmonary arterial endothelial cells (hPAECs) were purchased from ScienCell Research Laboratories (Carlsbad, CA, USA). hPASMCs and hPAECs were maintained at 37 °C in a humidified incubator containing 21% O_2_, 5% CO_2_ in air (referred to as normoxic conditions). For hypoxia experiments, cells were transferred into 5% O_2_, 5% CO_2_ and balanced nitrogen, and harvested at the indicated time.

### RNAi

Cells were seeded in 12-well plates and transfected with siRNAs using Lipofectamine 2000 for 48 h following the manufacturer’s instructions. shRNAs were designed as previously described[33–35].

negative control: 5′-TTCTCCGAACGTGTCACGT-3′, mouse *hif1α*-1: 5′-GCAGACCCAGTTACAGAAACC-3′, mouse *hif1α*-2: 5′-CTGGACACAGTGTGTTTGATT-3′, mouse *Plk1*: 5′-GCAGCAGGAAACCTCTCAAAG-3′, human *HIF1α:* 5′-GCTGGAGACACAATCATAT-3′, human *PLK1*: 5′-AGATCACCCTCCTTAAATATT-3′, human *RELA*: 5′-GCCCTATCCCTTTACGTCA-3′.

The siRNAs and their controls were synthesized by Shanghai GenePharma (Shanghai, China).

### Flow cytometry analysis

After treatment with 50 nM BI 2536 or transfection with siPLK1 for 24 h, hPASMCs were analyzed with Annexin V-fluorescein isothiocyanate (FITC) and propidium iodide (PI) apoptosis assay kit (FAK011, Neobioscience, Shanghai, China) according to the manufacturer’s protocol. Briefly, 1 × 10^6^ cells were collected. After washed twice with PBS, cells were incubated with Annexin V-FITC and PI for 15 min at room temperature in the dark. Cells were then immediately analyzed with the BD Accuri C6 flow cytometer (BD Biosciences, San Jose, CA, USA).

### Chromatin immunoprecipitation

hPASMCs were grown on 15-cm plates with or without hypoxia (10% oxygen) for 24 h. Cells were then fixed with 1% (vol/vol) formaldehyde (final concentration) for 10 min. Cross-linking was stopped with addition of 0.125 M glycine (final concentration). The cells were washed with cold PBS and scraped into PBS. After spinning down and discarding supernatant, cells were resuspended in 1 mL Buffer A [100 mM Tris·HCl (pH 8.0), 10 mM DTT], incubated on ice for 15 min. The samples were resuspended by vortexing and incubated at 30 °C for 15 min. Cells were then lysed step by step in Buffer B [0.25% Triton X-100, 10 mM EDTA, 0.5 mM EGTA, 10 mM HEPES (pH 7.5)], Buffer C [200 mM NaCl, 10 mM EDTA, 0.5 mM EGTA, 10 mM HEPES (pH 7.5)], and Buffer D [50 mM Tris·HCl (pH 8.0), 10 mM EDTA, 1% SDS, 10 mM HEPES (pH 7.5) and a protease inhibitor mixture; Roche] for 5 min each on ice. Cells were then sonicated to shear DNA under conditions established to ensure that the DNA fragments were between 500 and 1500 bp. After removal of insoluble material by centrifugation, supernatant was diluted in ChIP dilution buffer [1.2% Triton X-100, 1.2 mM EDTA, 180 mM NaCl, and 15 mM Tris·HCl (pH 8.0)]. The samples were precleared with 20 μL of blocked Protein A/G Agarose 50% slurry (Santa Cruz, sc-2003) for 1 h at 4 °C. 200 μL (20%) from supernatant was added with 6 μL 5 M NaCl (final concentration: 300 mM) and incubated overnight at 65 °C to determine DNA concentration and fragment size. The rest of supernatant was immunoprecipitated with polyclonal anti-HIF1α antibody, or polyclonal p65 antibody, or rabbit control IgG overnight at 4 °C on a rotating wheel. The immunocomplexes were recovered by addition of 20 μL of Protein A/G Agarose 50% slurry for 1 h at 4 °C. The samples were then sequentially washed for 5 min twice in ChIP low-salt wash buffer [0.1% SDS, 1% Triton X-100, 2 mM EDTA, 20 mM Tris·HCl (pH 8.0), and 150 mM NaCl], ChIP high-salt wash buffer [0.1% SDS, 1% Triton X-100, 2 mM EDTA, 20 mM Tris·HCl (pH 8.0), and 500 mM NaCl], ChIP LiCl buffer [250 mM LiCl, 1% Nonidet P-40, 1% deoxycholate, 1 mM EDTA and 10 mM Tris·HCl (pH 8.0)] and TE buffer [10 mM Tris (pH 8.0) and 1 mM EDTA]. The immunocomplexes were extracted twice with the elution buffer containing 1% SDS and 0.1 M NaHCO_3_. The eluates were pooled and the cross-linking was reversed by addition of 200 mM NaCl (final concentration) and incubation overnight at 65 °C. DNA was purified by phenol–chloroform extraction twice, followed by ethanol precipitation. DNA were resuspended in 40 μL of MilliQ water and amplified by real-time PCR.

SYBR Green PCR analysis with iQ SYBR Green Supermix was processed on an iCycler (Bio-Rad). For all primer sets, identical thermocycler conditions were used: stage 1, 3 min at 95 °C; stage 2, 40 cycles, with 1 cycle consisting of 15 s at 95 °C, 30 s at 60 °C, and 30 s at 72 °C. Each PCR was performed in duplicate. Because PCR amplification was being monitored by SYBR Green, the absence of nonspecific amplification was determined by analyzing the melting curve of the PCR amplification products. Data collected were analyzed using Microsoft Excel and plotted using GraphPad Prism software. Ten percent of the amount of chromatin was used for the input in our assay, the input dilution factor was therefore 10 times. We used delta-delta-CT method to analyze the fold enrichment following RT-PCR. Briefly the calculation was (1) DeltaCt = Ct ChIP − (Ct input – Log2(Input Dilution Factor)), (2) delta-deltaCt = deltaCt – Ct IgG, (3) old Enrichment = 2^ (− delta-deltaCt) [34].

Primers were designed within the two predicted binding regions (PBR of HIF1a and PBR of RELA) and at 1–2 kb downstream of the transcription start site as no-binding control (NBR) using Primer Premier 5. The primers indicated are:

*RELA*-PBR-F: 5′-GGGCGTCCGTGTCAATCAGGTT-3′, *RELA*-PBR-R: 5′-GCGGGCGGGTTTGGATTTTAA-3′, *HIF1α*-PBR-F: 5′-GGCGGGAGGATTGCTTGAACC-3′, *HIF1α*-PBR-R: 5′-CCCCCCACGCCTTTTTTTTGT-3′, *RELA-*NBR-F: 5′-GGGCCCCAGAGTTAGAGTGAGTGTC-3′, *RELA*-NBR-R: 5′-GCACCAGTCTCCCAGCCACAAC-3′, *HIF1α*-NBR-F: 5′-GCCCTCTCCTTCCCACCCACAGT-3′, *HIF1α*-NBR-R: 5′-GCCCAGCTTGAGGTCTCGATGAATA-3′.

### Animal studies

All animal experiments were approved by the Animal Research Committee, Institute of Basic Medical Sciences, Chinese Academy of Medical Sciences & Peking Union Medical College. Experiments were designed and performed in accordance with the relevant guidelines and regulations [36, 37]. At the end of animal experiments, all mice were anaesthetized with 2% pentobarbital sodium (50 mg/kg i.p.) and the lungs and hearts were quickly removed for evaluation.

### Hypoxia-induced pulmonary hypertension

#### Induction

Normobaric hypoxic chambers were maintained with approximately 6 L/min flow of hypoxic air (10% O_2_ and 90% N_2_) and were opened twice a week for cleaning and replenishment of food and water. To assess the influence of hypoxia on PLK1 expression, 8-week-old male C57BL/6 mice (Vital River Laboratories, Beijing, China) were randomly assigned to be kept in either 10% oxygen or in room air (21% oxygen) [38]. At 0, 4, and 6 weeks, 15 mice in each group were sacrificed for analysis. To examine the effect of PLK1 on PH, 8-week-old male C57BL/6 *Plk1*^+*/−*^ mice and *Plk1*^+*/*+^ male littermates (provided by Xiaochun Yu and Junjie Chen [39]) were kept in 10% oxygen for 0, 4, or 6 weeks. Fifteen mice per group were examined at each time point. In addition, 8-week-old male C57BL/6 mice were kept in 10% oxygen for 2 weeks, and randomly assigned to receive a tail-vein injection of either PLK1 inhibitor BI 2536 (50 mg/kg body weight, once per week, n = 15) [24], or 0.9% normal saline (vehicle, n = 15).

#### Hemodynamic measurements and pulmonary angiography of hypoxia-induced pulmonary hypertension

Mice were anesthetized with Ketamin (100 mg/kg) and fentanyl (0.05 mg/kg) intraperitoneally as described [40]. A Millar 1.4F Mikro-Tip catheter (SPR-671, Houston, TX, USA) was inserted via the right external jugular vein to record right ventricular systolic pressure (RVSP) (closed chest) at each time point. Heart was removed and RV free wall was dissected from the left ventricle (LV) plus septum (S) and weighed separately. The degree of right ventricular hypertrophy was determined from the ratio of RV/LV + S. To assess the effect of BI 2536 on LV function and cardiac output [40, 41], Millar pressure–volume microtip catheter was placed into LV through right carotid artery. Hemodynamic parameters were collected and analyzed with PVAN software (Millar Instrument) and the mice were sacrificed for assessment of right ventricular hypertrophy. Systemic blood pressure was measured from aortic pressure traces. Yellow Microfil solution was prepared and used following Dr. Patricia Thistlethwaite laboratory’s protocol for pulmonary angiography [40]. Vascular branches and junctions in the angiograms of lungs were quantified [42].

### Hypoxia- and Sugen5416-induced pulmonary hypertension

#### Induction

Sugen5416 is a potent, selective VEGFR (Flk-1/KDR) inhibitor with an IC50 of 1.23 μM [43] and a half-life of 30 min [44]. Twenty 8-week-old C57BL/6 mice under 10% O_2_ were subcutaneously injected with Sugen5416 (20 mg/kg body weight, once per week) for 3 weeks and then continually kept in hypoxia chamber (BioSpherix, NY, USA) for another 3 weeks. During the last 3 weeks, these mice were treated randomly with either 0.5% hydroxyethyl cellulose (vehicle) (n = 10) or BI 6727 (15 mg/kg body weight biweekly) (n = 10) by gavage. Twenty control mice were subcutaneously injected with DMSO once per week for 3 weeks under normoxia. These mice were then treated randomly with either vehicle (n = 10) or BI 6727 (15 mg/kg body weight biweekly) (n = 10) by gavage for 3 weeks [45].

#### Hemodynamic measurements of hypoxia- and Sugen5416-induced pulmonary hypertension

Firstly, hemodynamic measurements were performed with echocardiography. Echocardiography was evaluated with the high-resolution Micro-Ultrasound system (Vevo 770, VisualSonics, Toronto, Ontario, Canada). Mice were anesthetized with 1.5% isoflurane. Right ventricle wall thickness during diastole (RVWTD) were obtained from parasternal short axis view at papillary muscle level using M-mode. Pulmonary arterial (PA) peak flow velocity and PA acceleration time/ejection time (AT/ET) were obtained from parasternal short axis view at aortic valve level using pulsed Doppler mode [46–51]. The data were measured using VisualSonics Vevo 770 analysis software with a cardiac measurement package based on the average of at least 3 cardiac cycles. Secondly, a 22-gauge needle connected to the PowerLab system (AD Instruments, Sydney, Australia) was inserted into the right ventricles of the mice to record RVSP using Chart program in PowerLab system. Lastly, mice were sacrificed for assessing right ventricular hypertrophy (RV/LV + S).

### Histological analyses

After hemodynamic measurements and exsanguination, the left lungs were fixed for histology in 10% neutral buffered formalin, and the right lungs were snap-frozen in liquid nitrogen. H&E and immunofluorescence (IF) staining experiments were conducted as previously described [33]. Nuclei were labeled by DAPI staining. Images were captured with a Nikon microscope digital camera system.

### TUNEL assay

Mouse lungs were fixed, embedded and sectioned into 5 μm thick slices. TdT-mediated dUTP Nick-End Labeling (TUNEL) assay was performed using TUNEL Apoptosis Assay Kit (C1086, Beyotime, Beijing, China) according to the manufacturer’s instruction. Nuclei were labeled by DAPI staining. Images were captured with a Nikon microscope digital camera system.

### Determination of mRNA expression by qRT-PCR for Fig. 2

Total RNA was extracted from cells and reversely transcribed, cDNA was then used as template in a quantitative PCR reaction. Oligonucleotide primers were synthesized to detect *Plk1* with *Actb* as internal control. The primers used were as follows:

mouse *Hif1α* Forward: 5′-GGCGGCGAGAACGAGAAG-3′, mouse *Hif1α* Reverse: 5′-GACCACCGGCATCCAGAAG-3′; mouse *Plk1* forward-1: 5′-CTTCGCCAAATGCTTCGAGAT-3′, mouse *Plk1* reverse-1: 5′-TAGGCTGCGGTGAATTGAGAT-3′; mouse *Plk1* forward-2: 5′-CTAGCACACCAACACGTCGTA-3′, mouse *Plk1* reverse-2: 5′-ACCTCCAGATCCTCGTTCAGG-3′; mouse *Actb* forward: 5′-AGAGGGAAATCGTGCGTGAC-3′, mouse *Actb* reverse: 5′-CAATAGTGATGACCTGGCCGT-3′.

Primers were synthesized by Sangon Biological Engineering Technology & Services (Shanghai, China).

### Determination of mRNA expression by qRT-PCR for Fig. 6

Total RNA was extracted from lung and heart using Trizol method (Sigma-Aldrich, T9424). The first strand cDNA was synthesized from 1 μg of total RNA with HIScript-II Q RT SuperMix for qPCR (Vazyme, R223-01). Quantitative RT-PCR was performed on an iCycler IQ system (Bio-Rad) utilizing SYBR Green Master Mix (Transgen) according to the manufacturer’s instructions. The thermal cycle conditions were operated as 94 ℃ for 30 s, 94 ℃ for 5 s, 60 ℃for 1 min and a total of 40 cycles. Target mRNA was determined using GAPDH as the comparative cycle threshold method. All samples were run in duplicate. The primer sequences were listed below:

Gapdh: 5′-GGTTGTCTCCTGCGACTTCA-3′; 5′-GGTGGTCCAGGGTTTCTTACTC-3′; ANF: 5′-CACAGATCTGATGGATTTCAAGA-3′; 5′-CCTCATCTTCTACCGGCATC-3′; BNP: 5′-GAAGGTGCTGTCCCAGATGA-3′; 5′-CCAGCAGCTGCATCTTGAAT-3′; Collagen I: 5′-GAGTACTGGATCGACCCTAACCA-3′; 5′-ACGGCTGAGTAGGGAACACA-3′; Collagen III: 5′-TCCCCTGGAATCTGTGAATC-3′; 5′-TGAGTCGAATTGGGGAGAAT-3′; Il-1β: 5′-CTTCCCCAGGGCATGTTAAG-3′; 5′-ACCCTGAGCGACCTGTCTTG-3′; Il-6: 5′-GCTACCAAACTGGATATAATCAGGA-3′; 5′-CCAGGTAGCTATGGTACTCCAGAA-3′.

### Immunoblotting

Immunoblotting was conducted as described previously [52–54]. Tissue and cell proteins were extracted from SDS loading buffer with Protease and Phosphatase Inhibitor Cocktail (EDTA-Free) (P002, New cell and molecular, Suzhou, Jiangsu, China). The lysates were collected after centrifugation. After boiling for 10 min and cooling to room temperature, lysates were then subjected to immunoblotting. β-actin or alpha-smooth muscle-actin was used as the loading control. Signals were visualized with superenhanced chemiluminescence detection reagent.

### Statistical analyses

All data were analyzed using GraphPad Prism 7.0 (GraphPad software, San Diego, CA, USA). Quantitative data of mouse experiments were reported as median and interquartile ranges (IQR). Normally distributed data were assessed by two-tailed, unpaired Student’s *t*-test between two groups. One-way of variance (ANOVA), followed by Dunnett’s post hoc test, was used between multiple groups. Quantitative data of cell experiments were reported as mean ± SD, Excel software was used to analyze cell proliferation and quantitative real-time RT–PCR data. A *p* value of < 0.05 was considered significant.

## Results

### Enhanced PLK1 expression in mice with pulmonary hypertension

**Fig. 1:**
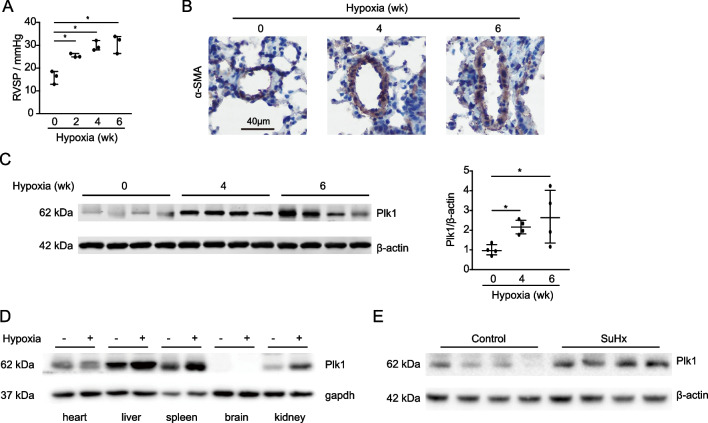
Enhanced PLK1 expression in mice with pulmonary hypertension. **A**–**C** Mice were exposed to hypoxia for up to 6 weeks. **A** Mouse RVSP. n = 3. **B** α-SMA immunohistochemical staining of mouse lungs. Scale bar, 40 μm. **C** Immunoblotting (left) and quantification (right) of mouse lungs. n = 4. **D** Immunoblotting of various organs from mice exposed to hypoxia (+) or normoxia (−) for 6 weeks. **E** Immunoblotting of lungs from the mice subcutaneously injected with Sugen5416 (20 mg/kg body weight, once per week) and kept in a hypoxia chamber at 10% oxygen for 3 weeks. ^*^P < 0.05

To investigate the role of PLK1 in PH, we checked the expression of PLK1 in mice with pulmonary hypertension. The averaged value of RVSP (ranging from 16 to 26 mmHg) is 21 mmHg in the 8-week-old male C57BL/6 mice [55]. RVSP, thickness of vascular smooth muscle and pulmonary Plk1 were increased over various periods of time under 10% oxygen (Fig. 1A–C). Plk1 levels were also higher in the heart, liver, spleen, kidney and muscle of the mice under hypoxia (Fig. 1D). In addition, Plk1 was boosted in the lungs of the mice exposed to 10% oxygen and Sugen5416 injection (SuHx) for 3 weeks (Fig. 1E). Taken together, hypoxia and SuHx induces PLK1 expression in mice.

### Hypoxia stimulates PLK1 expression through induction of HIF1α/NF-κB pathway

**Fig. 2:**
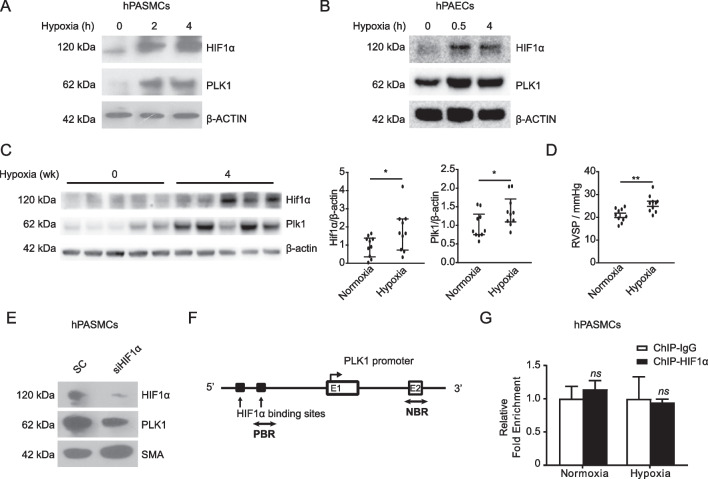
HIF1α indirectly induces PLK1 expression. **A**, **B** Immunoblotting of hPASMCs (**A**) and hPAECs (**B**) under hypoxia conditions (5% oxygen). **C**, **D** Mice were exposed to hypoxia (10% oxygen) or normoxia for 4 weeks. n = 10. **C** Immunoblotting (left) and quantification (right) of mouse lungs. **D** Mouse RVSP. **E** Immunoblotting of hPASMCs treated with or without siRNA against HIF1α. SC, control siRNA. **F** Schematic representation of the promoter region of human PLK1 gene. E1 and E2 designate PLK1 exons 1 and 2. Dark rectangles indicate predicted HIF1α binding regions; two-way arrows show the fragment amplified in ChIP real-time PCR analysis. The transcription start site is denoted by an arrow above the gene. PBR, predicted binding region; NBR, nonspecific binding region. **G** hPASMCs were cultured under hypoxia or normoxia for 24 h and collected for ChIP experiments. The target protein was immunoprecipitated with either anti-HIF1α antibody or IgG. Immunoprecipitated DNA was PCR-amplified for regions described in **F**. The data are plotted as fold enrichment with delta-delta-CT method. Representative data from two independent experiments are shown. n = 3. *P < 0.05; ns, not significant

Since HIF1α is a hypoxia-stabilized transcription activator, we reasoned that hypoxia-induced PLK1 expression might be mediated by accumulated HIF1α. Hypoxia (5% oxygen) induced time-dependent HIF1α and PLK1 protein elevation in hPASMCs and hPAECs (Fig. 2A, B). Both Hif1α and Plk1 were also increased in the lungs (Fig. 2C) along with elevation of RVSP in mice with hypoxia exposure (Fig. 2D). Depleting *Hif1α* compromised Plk1 in hPASMCs (Fig. 2E). We then checked whether HIF1α transcriptionally stimulated PLK1 expression. However, the potential binding site of HIF1α was more than 1 kb upstream of the transcription initiation site of PLK1 (Fig. 2F). Real-time PCR analysis of ChIP DNA demonstrated that HIF1α was not enriched in PLK1 promoter region, suggesting that PLK1 is not the direct transcriptional target of HIF1α (Fig. 2G).

**Fig. 3:**
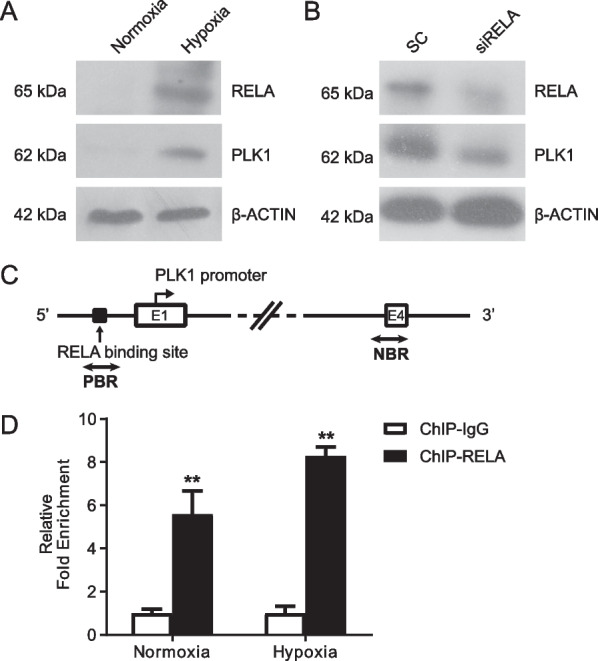
Hypoxia induction of RELA stimulates PLK1 transcription. **A**, **B** Immunoblotting of hPASMCs. **A** Cells were exposed to 5% oxygen for 24 h. **B** Cells were transfected with siRNA for RELA for 24 h and then placed under 5% oxygen for 24 h. **C** Schematic representation of the promoter region of human PLK1 gene. E1 and E4 designate PLK1 exons 1 and 4. The dark rectangle indicates predicted RELA binding region; two-way arrows show the fragment amplified in ChIP real-time PCR analysis. The transcription start site is denoted by an arrow above the gene. PBR, predicted binding region; NBR, nonspecific binding region. **D** hPASMCs were cultured under hypoxia or normoxia for 24 h and collected for ChIP experiments. The target protein was immunoprecipitated with either anti-RELA antibody or IgG. Immunoprecipitated DNA was PCR-amplified for regions described in **C**. The data are plotted as fold enrichment with delta-delta-CT method. Representative data from two independent experiments are shown. n = 3. *P < 0.05

There are several layers of cross-talk between HIF1α and NF-κB [14]. Taken the facts that RELA transcriptionally activates PLK1 in esophageal squamous carcinoma cells [35], we wondered whether HIF1α promotes PLK1 expression through induction of RELA. While hypoxia induced the expression of both RELA and PLK1, knocking down *RELA* reduced PLK1 expression in hPASMCs (Fig. 3A, B). The potential binding site of RELA is at 93 base pairs (bp) upstream of the transcription initiation site of PLK1 promoter region (Fig. 3C). Real-time PCR analysis of ChIP DNA demonstrated that RELA was well enriched in the promoter region of PLK1 gene in hPASMCs (Fig. 3D), indicating RELA as a direct activator of PLK1 expression.

### *Plk1* heterozygous knockout mice are partially resistant to hypoxia-induced PH

**Fig. 4:**
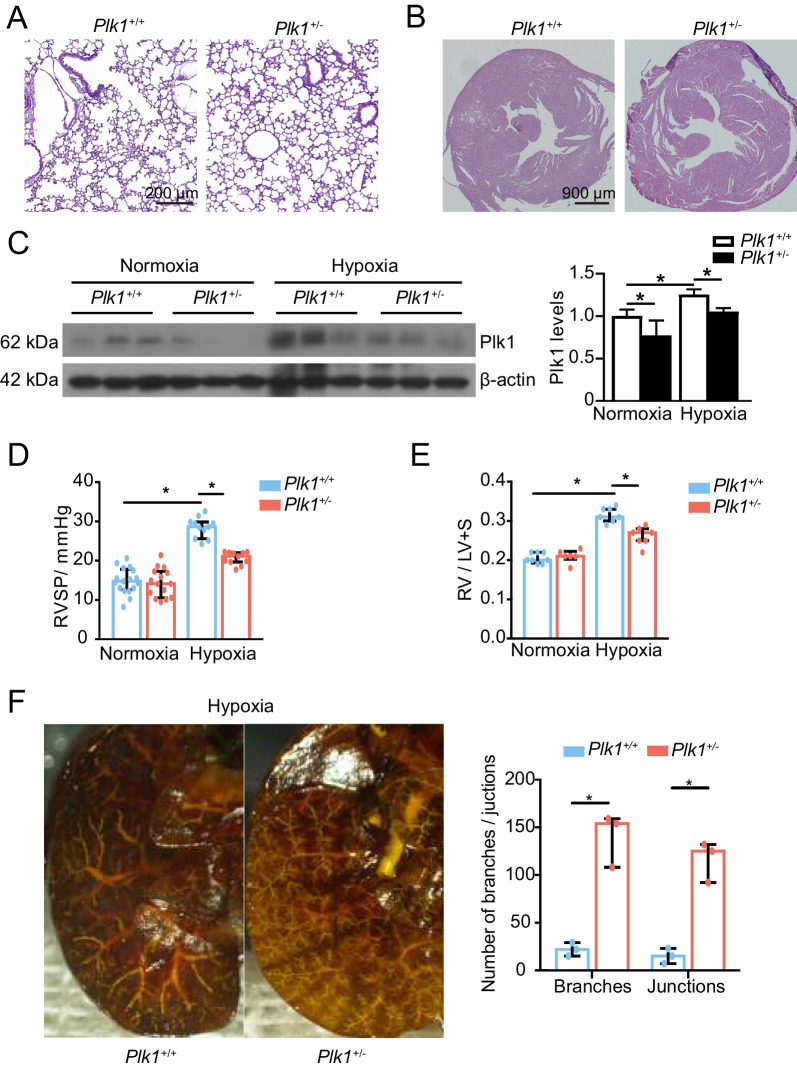
*Plk1 *heterozygous knockout mice are partially resistant to hypoxia-induced PH. **A**, **B** H&E staining of mouse lungs (**A**) and hearts (**B**) under normoxia. **C**–**F** *Plk1*^+*/*+^ and *Plk1*^±^ mice were exposed to normoxia or hypoxia (10% oxygen) for 4 weeks. **C** Immunoblottings (left) and quantification (right) of mouse lungs. n = 3. **D** Mouse RVSP. n = 11–17. **E** Mouse RV/LV + S. n = 6–8. **F** Representative pulmonary angiograms of *Plk1*^+*/*+^ and *Plk1*^±^ mice under 10% oxygen for 4 weeks (left). Numbers of vascular branches and junctions in angiogram were compared (right). n = 3. *P < 0.05

We assessed the development of hypoxia-induced PH in *Plk1* heterozygous knockout mice [39]. H&E staining showed that there were no obvious cardiac and pulmonary structural differences between *Plk1*^+*/*+^ and *Plk1*^±^ mice under normoxia (Fig. 4A, B). *Plk1*^±^ mice had decreased expression of Plk1 in the lungs under normoxic conditions (Fig. 4C). The lungs of WT mice contained higher level of Plk1 protein than those of *Plk1*^±^ mice under hypoxia (Fig. 4C). WT littermates presented with progressively elevated RVSP, with a median increment from 14.8 mmHg (interquartile ranges (IQR), 12.7–17.8) to 28.6 mmHg (IQR, 25.6–29.9) over 4 weeks of hypoxia exposure. In contrast, *Plk1*^±^ mice had mildly elevated RVSP, with a median increase from 14.2 mmHg (IQR, 10.5–17.3) to 21.1 mmHg (IQR, 19.7–22.1) (Fig. 4D). The ratio of the weight of right ventricle to left ventricle plus septum (RV/LV + S) increased from 0.20 (IQR, 0.19–0.22) to 0.31 (IQR, 0.30–0.33) in WT mice and from 0.21 (IQR, 0.20–0.22) to 0.27 (IQR, 0.25–0.28) in *Plk1*^±^ mice during a 4-week period of hypoxia conditioning (Fig. 4E). Since RVSP and RV/LV + S were not significantly different between WT and *Plk1*^±^ mice under normoxia (Fig. 4E), we conducted pulmonary angiography in hypoxia-conditioned WT and *Plk1*^±^ mice. As the pulmonary branches and junctions are destroyed, the surface area of alveoli for gas exchange would be reduced [56]. This can ultimately lead to increased right ventricular afterload and the development of pulmonary hypertension [57]. Angiograms showed blunting of the pulmonary vasculature with absence of peripheral artery filling in WT mice. In contrast, *Plk1*^±^ mice had a patent distal pulmonary vascular tree with more vessel branches and junctions (Fig. 4F). In addition, *Plk1*^±^ mice exhibited better cardiac output (CO) and ejection fraction (EF) compared with *Plk1*^+*/*+^ mice under hypoxia condition (Table 1). Enhanced PLK1 is thus essential in the development of hypoxia-induced PH.

**Table 1:** Left ventricular hemodynamics of WT and *Plk1*^±^ mice under hypoxia

Group	Heart rate (BPM)	Systolic pressure (mmHg)	Diastolic pressure (mmHg)	Mean pressure (mmHg)	CO (µL/min)	EF (%)
Plk1^+/+^ 0wk	378 ± 37	98 ± 12	85 ± 12	92 ± 11	7518 ± 218	46 ± 3
Plk1^+/+^ 4wk	462 ± 9	94 ± 11	78 ± 13	87 ± 11	5620 ± 277	33 ± 2
Plk1^±^ 0wk	364 ± 8	98 ± 10	81 ± 7	90 ± 7	7589 ± 209	45 ± 3
Plk1^±^ 4wk	414 ± 47	100 ± 12	78 ± 11	90 ± 12	6694 ± 388	38 ± 1

*wk* weeks since the beginning of hypoxia induction. n = 11–17

### PLK1 inhibitor BI 2536 attenuates chronic hypoxia-induced PH in mice

**Fig. 5:**
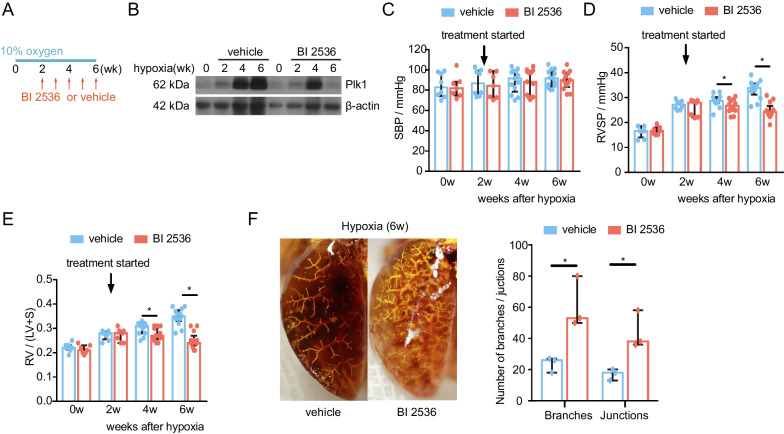
PLK1 inhibitor BI 2536 attenuates hypoxia-induced PH in mice. Mice were exposed to 10% oxygen up to 6 weeks. Treatment with intravascular PLK1 inhibitor BI 2536 or vehicle was started at the end of the second week. **A** Schematic schedules of PLK1 inhibitor BI 2536 treatment. **B** Immunoblotting of mouse lungs. **C** Mouse SBP. n = 7–13. **D** Mouse RVSP. n = 7–13. **E** Mouse RV/LV + S. n = 7–13. **F** Representative pulmonary angiograms of mice (left). Numbers of vascular branches and junctions in angiogram were compared (right). n = 3 ^*^P < 0.05

We used BI 2536, an ATP-competitive PLK1 inhibitor [24], to test the efficacy of PLK1 blockade in the treatment of PH. Mice were exposed to 10% oxygen (day 1) and then treated with vehicle or BI 2536, at the dose of 50 mg/kg body weight, once per week, from day 15 to day 42 (Fig. 5A). Inhibition of Plk1 in lung tissue was assessed by Western blotting at biweekly intervals during the course of BI 2536 administration. 4-week BI 2536 injection decreased Plk1 expression in the lungs of 6-week hypoxia-exposed mice (Fig. 5B). Systemic arterial blood pressure (SBP), RVSP and RV/LV + S were measured at 0, 2, 4, and 6 weeks. SBP was not significantly altered by hypoxia or PLK1 inhibition (Fig. 5C). Control mice developed pulmonary hypertension with elevated RVSP and RV/LV + S, while mice receiving BI 2536 had significant reductions in RVSP and RV/LV + S. The median RVSP decreased from 33.9 mmHg (IQR, 31.2–35.7) to 24.3 mmHg (IQR, 23.0–26.6) and the RV/LV + S reduced from 0.35 (IQR, 0.33–0.38) to 0.24 (IQR, 0.23–0.27) with 4 weeks of BI 2536 treatment (Fig. 5D, E). Even though the absolute values of RSVP varied among experiments conducted on different times (Figs. 1, 2 and 5), the trend of changes are similar. Angiogram analysis showed that BI 2536-treated mice had more branches and junctions of pulmonary vascular trees (Fig. 5F). In addition, BI 2536-treated mice exhibited increased CO and EF compared with vehicle-treated mice under hypoxia condition (Table 2). Collectively, PLK1 is essential for the development of PH and PLK1 inhibition is effective in the treatment of hypoxia-induced PH.

**Table 2:** Left ventricular hemodynamics of hypoxia-exposed mice treated with or without PLK1 inhibitor BI 2536

Group	Heart rate (BPM)	Systolic pressure (mmHg)	Diastolic pressure (mmHg)	Mean pressure (mmHg)	CO (µL/min)	EF (%)
C-0wk	378 ± 27	100 ± 13	82 ± 17	92 ± 14	7691 ± 214	46 ± 3
C-2wk	383 ± 54	98 ± 12	79 ± 15	89 ± 13	6795 ± 361	38 ± 1
C-4wk	354 ± 32	102 ± 11	83 ± 14	93 ± 13	5577 ± 284	33 ± 2
C-6wk	394 ± 8	97 ± 13	79 ± 16	89 ± 15	4884 ± 135	31 ± 4
D-4wk	432 ± 37	97 ± 13	76 ± 13	87 ± 13	6698 ± 279	38 ± 1
D-6wk	513 ± 8	97 ± 11	80 ± 8	90 ± 9	7562 ± 217	45 ± 3

C: vehicle-treated control mice; D: BI 2536-treated mice; wk, weeks since the beginning of hypoxia induction. n = 7–13

### PLK1 inhibitor BI 6727 mitigates Sugen5416/hypoxia-induced PH and right heart failure in mice

**Fig. 6:**
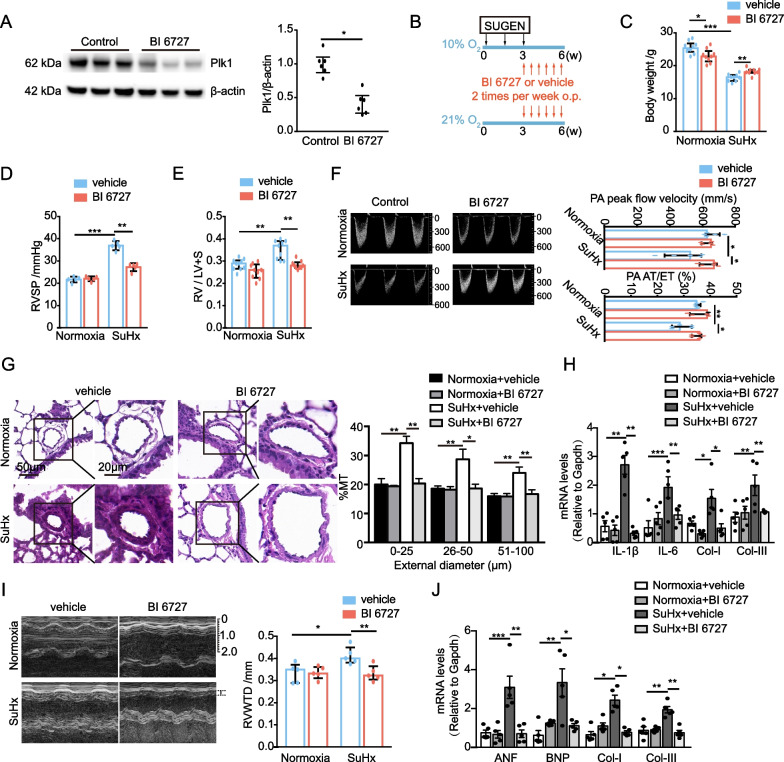
PLK1 inhibitor BI 6727 mitigates Sugen5416/hypoxia (SuHx)-induced PH and right heart failure in mice. **A** Immunoblottings (left) and quantification (right) of mouse lungs treated with PLK1 inhibitor BI 6727 or vehicle. n = 6. **B**–**I** Mice were kept in normoxic condition or 10% oxygen chamber for 6 weeks. During the first 3 weeks, mice under hypoxic condition were given Sugen5416. In the last 3 weeks, mice were treated with vehicle or PLK1 inhibitor BI 6727. **B** Schematic schedules of PLK1 inhibitor BI 6727 treatment. **C** The mouse body weights on the day of sacrifice. n = 10. **D** Mouse RVSP. n = 5. **E** Mouse RV/LV + S. n = 5. **F** Representative images illustrate changes of PA peak flow velocity (left). Echocardiography measurement of PA peak flow velocity and AT/ET ratio (right). n = 5. **G** Representative images (left) and quantification (right) of H&E-stained sections of small pulmonary arteries in mouse lungs. n = 3. **H** qRT-PCR of mouse lungs. n = 5. **I** Representative images (left) and quantification (right) of RVWTD. n = 5. **J** qRT-PCR of mouse hearts. n = 5. *P < 0.05

BI 6727 is also an ATP-competitive inhibitor of PLK1 and has better pharmacokinetic profile than that of BI 2536 [58–60]. While BI 2536 is administrated via intravascular injection, BI 6727 is given intragastrically. As expected [61], Plk1 was reduced by BI 6727 (Fig. 6A). Since Sugen5416/hypoxia (SuHx) causes severer PH by inducing pulmonary arterial remodeling, vascular occlusion and plexiform formation than hypoxia alone does in mice [62], we checked whether inhibition of PLK1 also reverses the pathogenesis of SuHx-induced mouse PH. Mice were subcutaneously administered Sugen5416 (20 mg/kg body weight, once per week) and kept in a hypoxia chamber for 3 weeks at 10% oxygen conditions. These mice in the hypoxia chamber were then randomly assigned to 2 groups for oral treatment with vehicle or BI 6727 (15 mg/kg body weight biweekly) for 3 weeks (Fig. 6B). Under normoxic condition, BI 6727 caused modest weight loss (Fig. 6C). However, BI 6727 slightly offset SuHx-induced severe weight loss in PH mice (Fig. 6C). Control mice had elevated RVSP and RV/LV + S while the mice with BI 6727 had significant reductions in RVSP and RV/LV + S (Fig. 6D, E). Echocardiography showed a significant decrease in peak velocity of blood flow and normalization of PA AT/ET ratio after BI 6727 administration (Fig. 6F). Histological analysis revealed that muscularization of peripheral pulmonary arteries was increased in control mice, whereas that was attenuated in BI 6727-treated mouse lungs (Fig. 6G). The expression of inflammatory genes (IL-1β and IL-6) and fibrotic genes (Collagen I and III) was reduced in the lungs of BI 6727-treated mice compared to that of control mice (Fig. 6H). Moreover, BI 6727 attenuated RVWTD, reduced the expression of hypertrophic genes (ANF and BNP) and fibrotic genes (Collagen I and III) (Fig. 6I, J), and restored CO and EF (Table 3) in SuHx-conditioned mouse hearts. Collectively, BI 6727 is effective in the treatment of SuHx-induced PH and right heart failure.

**Table 3:** Left ventricular hemodynamics of SuHx-administrated mice treated with or without PLK1 inhibitor BI 6727

Group	Heart rate (BPM)	Systolic pressure (mmHg)	Diastolic pressure (mmHg)	Mean pressure (mmHg)	CO (µL/min)	EF (%)
C-0wk	620 ± 78	94 ± 5	72 ± 6	79 ± 5	29,265 ± 1008	63 ± 6
D-0wk	584 ± 22	94 ± 4	73 ± 17	80 ± 4	29,216 ± 5619	62 ± 2
C-6wk	490 ± 51	95 ± 5	73 ± 6	80 ± 5	20,774 ± 2458	50 ± 3
D-6wk	579 ± 49	94 ± 3	73 ± 4	80 ± 3	23,888 ± 2033	61 ± 4

C: vehicle treated control mice; D: BI 6727 treated mice; wk, weeks since the beginning of SuHx induction. n = 10

### Suppression of PLK1 induces apoptosis

**Fig. 7:**
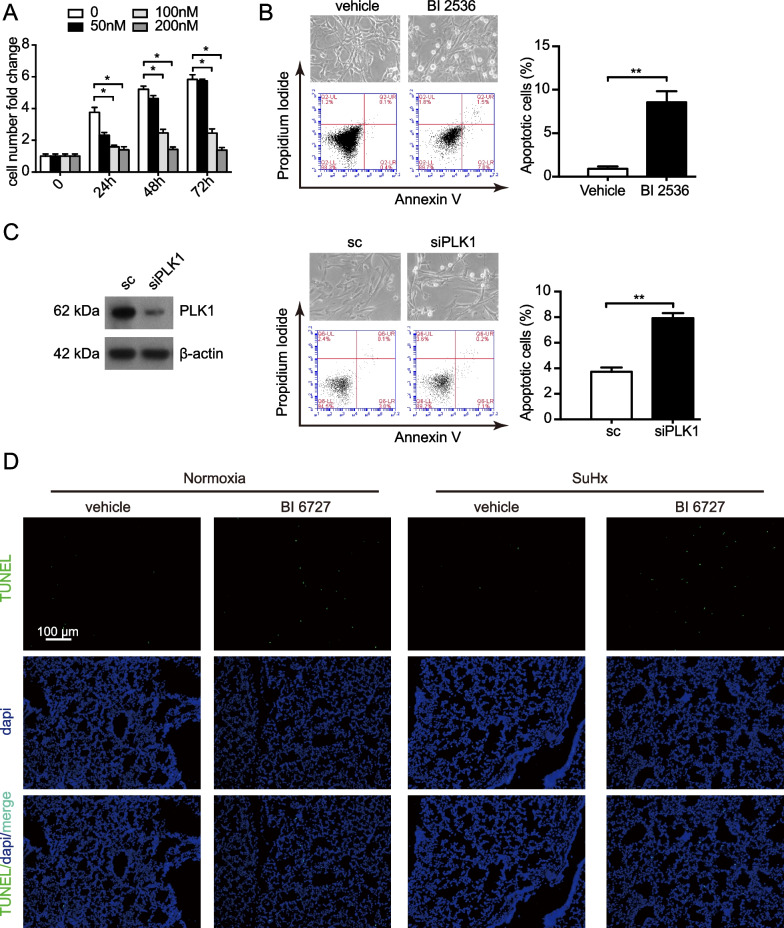
Suppression of PLK1 induces apoptosis. **A** The viability of hPASMCs treated with vehicle or BI 2536. n = 4. **B** hPASMCs were treated with or without 50 nmol/L BI 2536 for 24 h. Representative scatter plots of propidium iodide (PI) (y axis) vs Annexin V-FITC (x axis) of flow cytometry analysis (left). Quantification of apoptotic cells detected by flow cytometry (right). Bar chart showing average of 3 independent experiments. **C** Immunoblotting of hPASMCs transfacted with sc or siPLK1 for 24 h (left). Representative scatter plots of PI (y axis) vs Annexin V-FITC (x axis) of flow cytometry analysis (middle). Quantification of apoptotic cells detected by flow cytometry (right). Bar chart showing average of 3 independent experiments. **D** TUNEL staining (green) and dapi staining (blue) of mouse lungs treated with PLK1 inhibitor BI 6727 or vehicle. *P < 0.05

Proliferation of smooth muscle cells causes progressive obliteration of PA which in return increases resistance to blood flow [63]. We examined the effect of PLK1 inhibition on PASMCs. BI 2536 profoundly inhibited the viability of hPASMCs (Fig. 7A). Meanwhile, BI 2536 or *Plk1* knockdown, markedly induced apoptosis of hPASMCs, as determined by Annexin V-FITC analysis and cell morphology (Fig. 7B, C). BI 6727 increased apoptosis of lungs in SxHu-treated mice (Fig. 7D). The therapeutic impact of BI 2536 on hypoxia-induced pulmonary hypertension may involve induction of apoptosis on PASMCs.

## Discussion

PH is a devastating pulmonary arterial disease with high morbidity and mortality. In this study, we demonstrate that hypoxia stimulates PLK1 expression through induction of HIF1α and RELA. The elevation of PLK1 correlates with the rise of pulmonary arterial pressure in either hypoxia-induced or SuHx-induced PH mice. *Plk1* heterozygous mice are partially resistant to the development of hypoxia-mediated PH. Pharmaceutical inhibition of PLK1 attenuates PH. Therefore, PLK1 is essential for PH progression.

Hypoxia is a strong stimulus for PH. Alveolar hypoxia induces excessive proliferation of vSMCs that remodels pulmonary vascular wall [1, 36, 42, 45, 52–54, 58]. Since PLK1 is crucial in mitosis and PLK1 deficiency reduces cell proliferation/viability [11–13, 15–17], we speculated that PLK1 might play an essential role in the proliferation–apoptosis imbalance of PH. We noticed that hypoxia induced PLK1 expression both in vitro and in vivo. In quest of how hypoxia induced PLK1 expression, we focused on transcription factor HIF1α as hypoxia activates HIF1α and vascular smooth muscle HIF1α knockout abrogated the onset of PH in hypoxia-conditioned mice [64]. Even though depletion of HIF1α abrogated PLK1 expression, HIF1α did not bind directly to PLK1 promoter. NF-κB is also an essential transcription factor induced by hypoxia and is critical for hypoxic cellular responses. HIF1α and NF-κB have an extensive crosstalk under hypoxia [11, 65]. HIF1α activates RELA transcription [13]. NF-κB also stimulates HIF-1α transcription by binding to its promoter [66, 67]. Moreover, RELA interacts with HIF-1α to stimulate target gene expression [12]. RELA activated PLK1 transcription in esophageal squamous carcinoma cells [68]. We demonstrated that RELA was induced by hypoxia and a direct regulator of PLK1 expression in hPASMCs. Walmsley et al. reported that NF-κB was a hypoxia-regulated and HIF-dependent target. Depletion of HIF-1α reduced NF-κB signaling [69]. Therefore, hypoxia induces PLK1 expression likely through HIF1α-NF-κB signaling cascade.

To test the potential role of augmented PLK1 in the pathogenesis of hypoxia-induced PH, we checked the development of PH in *Plk1* heterozygous knockout mice as homozygous *Plk1* deficient mice were embryonic lethal. *Plk1* heterozygous knockout mice were resistant to hypoxic induction of PH. PH is a lethal respiratory disease without effective treatment [70]. As PLK1 inhibitors displayed antitumor efficacy and good tolerability in various human cancer xenograft models as well as in clinical trials, we proposed that PLK1 was an attractive target for the treatment of PH. We treated hypoxia-induced PH with PLK1 inhibitor BI 2536 and SuHx-induced PH with PLK1 inhibitor BI 6727 in mice. Both PLK1 inhibitors attenuated the development of PH. Therefore, PLK1 is essential for the development of PH and druggable for the treatment of PH.

Enhanced vSMC proliferation and attenuated apoptosis are the major features of pulmonary vascular remodeling during the development of PH. Excessive proliferation and/or too little apoptosis may contribute to sustained pulmonary vasoconstriction and excessive pulmonary medial hypertrophy in PH. As PLK1 inhibitors perturbed mitotic progression, rapidly dividing cells are more sensitive to PLK1 inhibition. Here, PLK1 inhibitor BI 2536 reduced the viability and induced apoptosis of PASMCs. BI 6727 increased apoptosis of lungs in SxHu-treated mice. These findings are consistent with what observed in PH-PASMCs exposed to siPLK1 or PLK1 inhibitor BI 6727 [71]. Induction of apoptosis and reduction of viability may contribute to the therapeutic effect of PLK1 inhibitor on PH.

In summary, hypoxia stimulates PLK1 overexpression through activation of HIF1α and RELA. Augmented PLK1 plays essential roles in the development of hypoxia/SuHx-induced PH. As inhibition of PLK1alleviates mouse PH, targeting PLK1 is therefore feasible for the treatment of PH. Due to the nature of largely animal study, these findings could not be directly extrapolated to PH patients. All these laboratory findings thus warrant validation from clinical trials of PLK1 inhibitors on PH.

## Data Availability

Data sharing not applicable to this article as no datasets were generated or analysed during the current study.
